# Vesico-ureteric reflux in children and young people undergoing kidney transplantation

**DOI:** 10.1007/s00467-022-05761-5

**Published:** 2022-10-24

**Authors:** Ian K. Hewitt, Giovanni Montini, Stephen D. Marks

**Affiliations:** 1 Perth Children’s Hospital, Monash Avenue, Nedlands, WA Australia; 2 Pediatric Nephrology, Dialysis and Transplant Unit, Fondazione IRCCS Ca’ Granda, Ospedale Maggiore Policlinico, via della Commenda 9, Milan, Italy; 3 Department of Clinical Sciences and Community Health, University of Milano, Milan, Italy; 4 Department of Paediatric Nephrology, Great Ormond Street Hospital for Children, NHS Foundation Trust, London, UK; 5 NIHR Great Ormond Street Hospital Biomedical Research Centre, University College London Great Ormond Street Institute of Child Health, London, UK

**Keywords:** Urinary tract infection, Vesico-ureteric reflux, Kidney transplant, Children, Adolescents

## Abstract

Vesico-ureteric reflux (VUR) into transplanted kidneys in children and young people is a common occurrence, found in 19 to 60% of those who had an anti-reflux procedure and up to 79% in the absence of such a procedure. While VUR is unlikely to be of concern without evidence of symptomatic urinary tract infections, less certainty exists regarding outcomes when the VUR is associated with urinary tract infection (UTI) and transplant pyelonephritis. Issues explored will include additional risk factors that might predispose to UTI, any effect of pyelonephritis on acute and long-term kidney allograft function and practical strategies that may reduce the prevalence of infection.

## Introduction

Vesico-ureteric reflux (VUR) into native kidneys, particularly the higher grades with dilatation of the ureters, is recognised as a risk factor for children and young people developing urinary tract infections (UTI) and predisposes to ascending infection with acute pyelonephritis (APN). This may result in some scarring, although function is largely preserved when this occurs in healthy kidneys. Prior to adequate antenatal ultrasound, poorly functioning kidneys, often in association with dilating VUR, were thought to be the consequence of pyelonephritic scarring of the immature kidney, which was termed “reflux nephropathy”. It is now recognised that children with chronic kidney disease in association with VUR most likely represent congenital anomalies of the kidney and urinary tract (CAKUT). VUR into transplanted kidneys raises additional concerns beyond APN and scarring in the allograft, an organ at risk of rejection prevented only by aggressive immunosuppression [1–3]. Febrile UTI after transplantation is common and has the potential to cause issues with transplant acute kidney injury and can cause damage and reduced function to the transplanted kidney in the longer term. It is highly likely that VUR into the transplanted kidney per se is common and not a concern in the absence of symptomatic urinary tract infection, similar to the situation where VUR occurs into native kidneys. The issues to consider are how common is VUR to the paediatric allograft, is persistent VUR to the native kidneys an issue, how common are post-transplant UTI and APN (and what are the predisposing factors to their occurrence), do they affect allograft survival, and if so, what preventative measures can be taken?

## Prevalence of transplant VUR

The prevalence of VUR to the paediatric allograft is variable in the literature and dependent upon whether an anti-reflux procedure is performed as well as the type of procedure. While most authors investigate VUR following urinary tract infections (UTIs), only four report routine post-transplant voiding cysto-urethrography (VCUG) for three cohorts of patients [4–7]. The prevalence of VUR ranged from 19 to 60% in those who had an anti-reflux procedure and up to 79% in the absence of such a procedure. Ranchin et al. described 65% (55 of 85) children who underwent a routine VCUG a median of 8 months post-surgery over a 5-year period. All but one child with a uretero-ureteral anastomosis had an anti-reflux implantation of the allograft ureter at the time of transplantation, despite which 60% (33 of 55) had VUR to the allograft, 17 of whom had ureteric dilatation [4]. Fontana et al. described 73 children who underwent a routine VCUG 6 months post-transplantation. All had the same Lich-Gregoir anti-reflux procedure with 34% (25 of 73) demonstrating VUR to the allograft (four patients with Grade I and seven patients each with grades II, III, and IV) [7]. The remaining two papers describe the same single-centre cohort. Those in whom an anti-reflux procedure was performed had a 19% (7 of 37) prevalence of VUR while in the absence of such a procedure the prevalence of VUR was 79% (15/19) [5, 6]. The high prevalence of VUR where an anti-reflux procedure has been performed likely reflects the preference for the Lich-Gregoir technique, as the ureter and bladder wall intra-mural component are kept short to ensure adequate blood supply at the anastomosis [8]. Furthermore, it is common to leave a temporary stent in situ that interferes with normal peristalsis and distends the vesico-ureteric junction [8]. Therefore, we can conclude that VUR to the transplanted kidney is common, even when the vesico-ureteral anastomosis entails an anti-reflux procedure. Although we believe that an anti-reflux procedure is appropriate, we acknowledge the limitations of the procedure with the less effective Lich-Gregoir technique being preferred to avoid necrosis or obstruction of the ureter.

## Prevalence of post-transplant native VUR

The prevalence of VUR to the native kidneys following paediatric kidney transplantation (KTx), like that to the allograft, is variable and dependent upon causation of kidney failure in the population transplanted. Those transplanted following kidney failure due to glomerular or tubulointerstitial diseases would be expected to have a lower incidence and severity of VUR compared with those transplanted due to CAKUT, the latter incidence further complicated by nephrectomy or anti-reflux surgery performed pre-transplantation. The one paper describing routine post-transplant VCUG where VUR to the native kidneys is reported quoted a prevalence of 15% (8 of 55 cases) [4].

## Post-transplant UTI risk

The risk of UTI following KTx in children and that of APN (usually defined as a febrile UTI in the absence of acute allograft rejection) has been addressed in at least four prospective studies [3, 9–11]. Urinary tract infections remain the commonest bacterial infection in paediatric kidney transplant recipients (pKTR) [12]. Weigel et al. described the largest and most comprehensive paediatric cohort, a multicentre prospective observational study that enrolled 98 pKTR aged ≤ 18 years who completed 2 years of follow-up with data recorded on febrile UTI (fUTI). In this cohort 39% of children had at least one fUTI post-transplant. Pre-transplant fUTI was more frequent in patients with CAKUT compared to those without CAKUT (38.7% vs. 12%; *p* = 0.005); however, no significant difference was noted after KTx (48.7% vs. 32.2%, *p* = 0.14) [9]. A further study of infectious complications in a cohort of 36 consecutive KTx patients aged 2–17 years followed for a median of 13.5 months reported UTI in 78% (28 of 36) of whom 19% (7 of 36) experienced fUTI [11]. The two remaining prospective studies reported combined results on children and adults. Rivera-Sanchez et al. reported a prospective observational study of UTI post-transplant in 52 patients aged 11–47 years. Urine cultures were performed every three days in hospital and weekly on discharge: 37% (19 of 52) developed a UTI within 75 days, although no age breakdown was given, and asymptomatic bacteriuria is not clinically relevant [10]. Patel et al. reported a multicentre randomised controlled trial (RCT) of early (day five) vs. late (around six weeks) transplant ureteric stent removal following KTx with the endpoint being the frequency of UTI during the 3 months post-transplant. The incidence of UTI was 7.6% (6 of 79) in the early compared with 24.6% (31 of 126) in the late group [3]. The duration of follow-up varied widely in the different studies making comparisons difficult; however, given a sufficient study duration, up to 78% experienced a UTI and 39% at least one fUTI.

## Risk factors for post-transplant UTI

The risk factors for developing a fUTI in the allograft is addressed in detail in the well-conducted Weigel et al. study [9]. Gender did not influence the frequency of infection; however, fUTI had an earlier peak in males (aged 1–18 (median 4) years of age) vs. females (aged 1–18 (median 13.5) years of age) (*p* = 0.02), with the mean age at transplant no different. Factors that did increase the frequency of fUTI included a history of fUTI prior to KTx (*p* = 0.001) and boys having diagnosis of posterior urethral valves (*p* = 0.004), while CAKUT as a group of disorders (*p* = 0.14), the type of anti-reflux procedure (*p* = 0.87) or the use of transplant ureteric stents (*p* = 0.33) did not affect the rate significantly. Patel et al. demonstrated that late vs. early removal of transplant ureteric stents following KTx increased the risk of UTI (24.6% vs. 7.6%; *p* = 0.004) in a cohort of 212 KTx recipients, aged 2–75 years of whom 37 were aged 0 to 16 years inclusive. The UTI rate was lower in the paediatric early removal group, although one of the exclusion criteria for this study was children with hostile bladders.

VUR into the native kidneys as a risk factor for UTI is addressed directly in one paper where routine post-transplant VCUG was performed on the entire study cohort (55 pKTR aged under 17 years of age) [4]. Of the eight (15%) children with VUR to the native kidney, three experienced at least one episode of APN (two of whom had no VUR to the graft). The number of children with VUR to the native kidneys was overshadowed by the much higher 60% (33 of 55) incidence of VUR to the allograft, such that no conclusions can be drawn as to its impact. Basiri et al. indirectly addressed the problem of reflux to the native kidneys, as a risk factor for UTI, in a retrospective study of 29 of 207 (14%) children transplanted from 1984 to 2003 with a history of VUR [13]. Patients were divided into two groups: 12 with VUR corrected prior to surgery and 17 with VUR who did not undergo anti-reflux surgery; 36 pKTR without VUR at cystography served as controls [13]. Pre-transplant correction of VUR did not result in a significant decrease in incidence of fUTI post-transplant when compared with those in whom VUR was uncorrected. However, both groups with VUR, whether corrected surgically or not, had a higher incidence of fUTI post-transplant [13]. The data are interesting, although they need to be interpreted with caution given that the paper does not report the incidence of VUR to the allograft for the 207 children transplanted, a potential confounder, as well as the number of transplanted children investigated for VUR being small.

With a single prospective observational study analysing risk factors for occurrence of paediatric post-transplant fUTI in any detail [9], it is apparent that larger, appropriately conducted studies are required. It does appear that the prevalence of fUTI post KTx is high regardless of the presence or absence of VUR into the allograft. It appears related to the presence of CAKUT, raising the possibility that bladder and bowel dysfunction may play a role with particular regard to pKTR with posterior urethral valves. The role of immunosuppression in predisposing to fUTI is less certain given that it does not play a significant role in occurrence of APN in native kidneys [14].

## Post-transplant kidney allograft scarring

The risk of allograft scarring following APN has been rarely addressed in appropriate well-designed studies, with most knowledge derived from the experience in native kidneys. It was thought that the “immature kidneys” of younger children were particularly prone to scarring following APN. Prospective RCTs demonstrated that APN in native kidneys was more common in children less than 2 years of age [15]. One paper evaluated scarring due to APN in 287 children aged from 2 months to 7 years abstracted from two prospective RCTs, found no significant difference in the risk or severity of scarring related to age [16, 17]. Many clinicians now advocate performing baseline DMSA scans 3 months post-transplant in those patients with hostile bladders to allow comparison with future DMSA scans after developing UTI [2]. Scarring due to APN is now well recognised in adult and paediatric kidneys, with at least one paper demonstrating the age of the donor kidney was not a variable in the propensity for scarring following UTI in pKTR [2]. The retrospective observational study was of 30 pKTR who had an early (within 2 weeks of function) and late (at least 1 year post-KTx) DMSA scan that was delayed by > 3 months in the event of any UTI. New focal defects occurred in 37% (11 of 30), scarring occurred in 53% (10 of 19) with a documented UTI and 9% (1 of 11) without a documented UTI (*p* = 0.02). While the occurrence of UTI in the presence of VUR led to scarring in 69% (9 of 13) of children, the absence of combined VUR and UTI led to scarring in only 1 of 14 children (7%) (*p* = 0.001). This child had VUR without a documented UTI. It is important to note that the manuscript gives no breakdown of fUTI. The 37% incidence of new focal defects on the late DMSA scan is higher than the 15% prevalence of native kidney scarring following a fUTI reported in a systematic review [18]. It is important to acknowledge that the patient number was small, the study retrospective, on a cohort of patients presumably at higher risk, and that transient defects in isotope uptake on DMSA can persist well beyond 3 months such that a delay of 6 to 12 months is recommended before stating any photopenic defect demonstrates permanent scarring. Therefore, it is not possible to be precise regarding the risk and severity of allograft scarring based on a single retrospective study.

There is also a retrospective study in adults where the authors identified 18.2% (56 of 307) adult KTR who had more than three UTI per annum beyond 6 months post-transplant [19]. The study cohort consisted of 32 KTR who underwent a DMSA scan and a VCUG (24 declined to be investigated). Forty-seven (15 of 32) had evidence of VUR (three, nine and three with grade II, III and IV VUR, respectively). Scarring was seen in 75% (24 of 32) including 87% (13 of 15) with VUR, but also 65% (11 of 17) of those without evidence of VUR. This may be due to VUR being a dynamic and not a constant phenomenon and KTR may have VUR without evidence of VUR at time of VCUG.

## Acute pyelonephritis involving the allograft: effect on kidney allograft survival and function

APN represents a significant insult to the allograft. Of concern, does it result in any short- or long-term adverse effects? In a prospective, multicentre observational study of fUTI in 98 pKTR aged 1 to 18 years, followed over 24 months, graft function declined acutely (*p* < 0.001), but was no different compared to patients without fUTI after 2 years [9]. Given that larger numbers might be required to determine any long-term detrimental effect, a further study of the US Renal Data System from 1996 to 2000 for composite inpatient and outpatient UTI early (less than 6 months) and late (from 6 to 36 months) post-transplant in children under 18 years identified 870 patients with Medicare as the primary funder. The risk of kidney allograft loss was elevated following early UTI (adjusted hazard ratio 5.47, CI 0.56–7.80; *p* < 0.001) but not after late UTI [20]. Three retrospective studies involving similar smaller numbers of children showed conflicting results, one demonstrating a faster deterioration in kidney allograft function at 4 years post-transplant in those with recurrent UTI [21] while two others demonstrated no correlation with kidney allograft function out to 5 years [22] or kidney allograft survival out to 10 years post-transplant [23]. In an adult study, Kaplan–Meier survival curves showed no difference in kidney allograft survival comparing those with and without scarring [19].

It is difficult to determine the role of APN as a consequence of VUR, as a risk factor affecting kidney transplant survival either through scarring or possibly predisposing to rejection. The US Renal Data System study is particularly interesting in relation to the increased kidney allograft loss only in the first 6 months. A number of factors could be at play, including delayed removal of stents [3], increased incidence of surgical interventions and complications, although this finding requires further research and clarification.

## Evidence for APN predisposing to acute rejection as a potential cause of kidney allograft loss

There are reports of viral infection, particularly CMV, predisposing to acute transplant rejection with the proposed cause being upregulation of Class II antigens secondary to the inflammation [24, 25]. Information on bacterial APN as a potential risk factor for transplant rejection is sparse. In adults there are anecdotal reports [26] and retrospective studies [27, 28] that highlight an increased risk of acute allograft rejection and decreased long-term function following APN. The evidence that any infection, viral or bacterial, predisposes to kidney allograft loss is tenuous. The few published studies, predominantly retrospective, that report an association between an infection, be it CMV or bacterial pyelonephritis, and graft rejection, do not prove causation.

## Bladder dysfunction and risks of UTI post-transplant

Patients with CAKUT have an associated risk of bladder dysfunction, which can predispose to UTI and APN post-transplantation. This risk can be attenuated when a multi-disciplinary team assessment is made collaboratively by paediatric nephrologists, urologists and transplant surgeons to ensure the bladder is safe, with good compliance and capacity. Children with obstructive uropathy, including posterior urethral valves, pelvi-ureteric and vesico-ureteric junction obstruction and those with known abnormal bladder urodynamics before KTx represent specific risk groups. Although information may be obtained from ward fill urodynamics assessment, which may show evidence of post-void residual requiring double voiding or clean intermittent urethral catheterisation, a more detailed video-urodynamics assessment via suprapubic catheter is usually required in the above at risk groups, to ensure if the bladder is truly safe for KTx.

When there is evidence of abnormal bladder urodynamics, the clinical team need to consider if the patient requires bladder augmentation (usually with formation of Mitrofanoff for easy catheterisation and bladder drainage) before or after KTx [29–31]. We advocate performing bladder augmentation before KTx to minimise the risks to the transplanted kidney, but this needs to be individualised as the surgery itself may mean that the patient moves from chronic kidney disease to requiring dialysis. When a patient is anuric and on dialysis, then performing bladder augmentation needs to be conducted shortly before KTx (easier if living donor available), due to the difficulties of cycling a dry bladder. It remains unclear if implantation of the ureter in an augmented bladder or a dysfunctional bladder represents a specific risk factor for the occurrence of post-transplant VUR.

Post-transplantation, it is important to continue to monitor patients and advise given the increased fluid demands of the KTx, that there is regular bladder emptying with recommendations of double voiding (or more regular catheterisation) if evidence of post-void residual on ultrasound screening. If there is evidence of asymptomatic bacteriuria (ABU) in an augmented bladder, then this likely reflects a colonised system and treating for ABU should be avoided, as this increases the chances of developing resistant bacteria, which can further cause UTI and APN. However, prompt review and management with early treatment with antibiotics should be advocated for all pKTR with fUTI. This can potentially minimise the risks of kidney scarring and damage. 

## Interventions: should they be considered?

A literature search for RCT of antibiotic prophylaxis for UTI in pKTR report a single paper that reported a benefit in the 3 months following transplantation, from a single intra-operative bladder instillation of amikacin in 200 patients randomised to amikacin vs. saline (25 vs. 49% incidence UTI, *p* = 0.0007) commenting that children and adults were included without any further breakdown of ages [32].

Expanding the search to RCT of antibiotic prophylaxis for UTI in KTR regardless of age detected a further three papers, one comparing a single vs. multiple peri-operative regimen of systemic antibiotic prophylaxis with subsequent UTI similar in both groups [33], a second randomising patients to bladder instillation of cephalothin or saline without any influence on outcome [34] and the third randomised 57 allograft recipients to three peri-operative doses of cefuroxamine and piperacillin vs. no treatment with UTI rate unaffected [35].

No prospective RCT has been reported on long-term low dose antibiotic prophylaxis post kidney transplant to prevent UTI. However, this is probably due to the fact that most centres employ co-trimoxazole antibiotic prophylaxis to their pKTR for prevention of *Pneumocystis jirovecii* pneumonia. The added benefit of this prophylaxis on the prevention of UTI post-transplant remains unknown. However, practically, this means that most clinicians aim to see if pKTR have had UTI during the phase of *Pneumocystis jirovecii* prophylaxis (usually 6 or 12 months) and decide if further urinary prophylaxis is required. The efficacy of antibiotic prophylaxis in the prevention of UTI and kidney damage as a consequence of APN is under consideration in the context of VUR to native kidneys [36, 37]. We believe in the absence of firm evidence that it is reasonable to employ antibiotic prophylaxis in those children, principally female, who experience more than three UTI per year.

There are considerations for other interventions to reduce post-transplant UTI which include ensuring that constipation is well treated. There has been emerging evidence that changes in the gastrointestinal microbiota may play a role in the development of bacterial infections after KTx [38]. However, there are theoretical concerns in utilising prebiotics and probiotics in immunosuppressed transplant recipients, although these may be reduced in ensuring they contain multiple strains.

There are some pilot data suggesting that immunisation against inactivated bacterial strains may reduce the incidence of UTI in adult KTR who have had recurrent UTI [39]. Although there were no safety concerns, this has only been conducted in 14 adult KTR who had three or more UTI episodes/year, with three subcutaneous injections of inactivated bacteria with only 12 months follow-up after immunisation. There are limited data on the prevalence of the bacterial spectrum in fUTI pKTR. The single prospective RCT in 55 children demonstrated the most common isolated microorganisms to be *Escherichia coli* (21%), *Enterococcus* (13%), *Staphylococcus* sp. (10%), *Klebsiella* (8%) and *Proteus* sp. (5%) [9].

## Conclusions

Children with CAKUT who have evidence of hostile bladders and VUR have an increased incidence of post-transplant UTI and APN. Although VUR into transplanted kidneys can be reduced due to the surgical method of ureteric anastomosis, it is highly likely that VUR into the transplanted kidney per se is common and not a concern in the absence of infection. Post-transplant APN can potentially damage a precious transplanted kidney. Therefore, it is important to reduce the number of symptomatic UTI and the impact they can have on future kidney allograft function and survival. Generally, an anti-reflux procedure at transplantation, a prompt antibiotic treatment of any symptomatic UTI and advising double voiding at micturition are practical strategies.

### Key summary points

1. Children with CAKUT who have evidence of hostile bladders with vesico-ureteric reflux (VUR) have an increased incidence of post-transplant UTI and APN.
2. Although VUR into transplanted kidneys can be reduced due to the surgical method of ureteric anastomosis, it is highly likely that VUR into the transplanted kidney is common and not a concern in the absence of infection.
3. Prompt review and management with early antibiotics should be advocated for pKTR who develop UTI after KTx to minimise the risks of kidney scarring and damage to a precious transplanted kidney which could impact on future kidney allograft function and survival.

### Multiple-choice questions (the answers to these questions can be found after the reference list)

1. What is the prevalence of transplant VUR in pKTR who have had an anti-reflux procedure performed at time of transplant?
  - 20–40%
  - 20–60%
  - 10–20%
  - 10–40%
  - 5–10%
2. When is the earliest time to perform DMSA scan after UTI to check for evidence of kidney scarring?
  - 2 weeks
  - 1 month
  - 6 weeks
  - 2 months
  - 3 months
3. Which clinical interventions may reduce the frequency of post-transplant UTI?
  - Double voiding
  - Early removal of transplant ureteric stent
  - Regular bladder emptying
  - Treatment of constipation
  - All of the above
